# COVID-19 and underlying health conditions: A modeling investigation

**DOI:** 10.3934/mbe.2021191

**Published:** 2021-04-30

**Authors:** Chayu Yang, Jin Wang

**Affiliations:** 1 Department of Mathematics, University of Florida, Gainesville, FL 32607, USA; 2 Department of Mathematics, University of Tennessee at Chattanooga, 615 McCallie Ave., Chattanooga, TN 37403, USA

**Keywords:** COVID-19 transmission, chronic conditions, data fitting

## Abstract

We propose a mathematical model based on a system of differential equations, which incorporates the impact of the chronic health conditions of the host population, to investigate the transmission dynamics of COVID-19. The model divides the total population into two groups, depending on whether they have underlying conditions, and describes the disease transmission both within and between the groups. As an application of this model, we perform a case study for Hamilton County, the fourth-most populous county in the US state of Tennessee and a region with high prevalence of chronic conditions. Our data fitting and simulation results quantify the high risk of COVID-19 for the population group with underlying health conditions. The findings suggest that weakening the disease transmission route between the exposed and susceptible individuals, including the reduction of the between-group contact, would be an effective approach to protect the most vulnerable people in this population group.

## 1. Introduction

COVID-19 has been a global pandemic for more than one year, with over 100 million cases reported throughout the world. In the United States (US) alone, COVID-19 already led to nearly 30 million cases and over half million deaths, as of early March, 2021. The elderly and those with chronic conditions have been among the most vulnerable groups for the COVID-19 infection [1, 2].

It is estimated that 22% of the global population, or 1.7 billion people, have at least one underlying health conditions that put them at higher risk for severe COVID-19 associated illness, and that 4% of the global population, or 349 million people, would require hospital admission if infected with COVID-19 [3]. A recent study conducted by CDC reports that among COVID-19 cases, the most common underlying health conditions are cardiovascular disease (32%), diabetes (30%), and chronic lung disease (18%). It is also found that among those with reported underlying conditions, hospitalizations were 6 times higher and deaths were 12 times higher compared to those without an underlying condition [4]. In another study, it is found that among 3,142 US counties, the median estimate of the prevalence of any of five underlying medical conditions (chronic obstructive pulmonary disease, heart disease, diabetes, chronic kidney disease, and obesity) associated with increased risk of severe COVID-19 infection among adults is 47.2%. Counties with the highest prevalences of these health conditions are concentrated in Alabama, Mississippi, Tennessee, and several other southeastern states [5].

The widespread presence of underlying health conditions plays a significant role in raising the numbers of severe COVID-19 infections and subsequent hospitalizations, in contributing to the disease-induced mortality rates, and in shaping the overall pattern of the COVID-19 epidemics. On the other hand, the quantitative relationship between the transmission and spread of COVID-19 and the underlying health conditions of the population remains unclear at present, which hinders our further understanding of COVID-19 dynamics and the design of effective control strategies to protect the most vulnerable [6, 7]. In this work, we propose to use mathematical modeling to study this relationship and to quantify the impact of chronic conditions on the COVID-19 transmission dynamics. Thus far, there have been a large number of mathematical, statistical and computational models developed to study the transmission and spread of COVID-19 and to forecast its epidemic development (see, e.g., [8–17] and references therein). However, to our knowledge, none of these models have been designed to investigate the effects of the chronic medical conditions on the incidence, prevalence and transmission of COVID-19 and associated severe illness.

Our aim is to develop a general modeling framework that can quantify the correlation between COVID-19 transmission and underlying medical conditions, and predict the specific numbers of the infected individuals with underlying conditions and those without such health conditions. To that end, we divide the host population into two groups, depending on whether or not they have underlying health conditions. Individuals within each group are classified into the susceptible, exposed, infected, hospitalized, and recovered compartments, where both the exposed and infected individuals are capable of transmitting the disease, and where the hospitalized compartment contains individuals with severe COVID-19 infection. Our model then describes the disease progression within each group as well as the cross-transmission of the disease between the two groups.

As a demonstration of our modeling work, we apply it to study the transmission of COVID-19 in Hamilton County, the fourth-most populous county in the US state of Tennessee. The total population of Hamilton County is 367,804 [18] and Chattanooga, the fourth-largest city in Tennessee, is its county seat. With several cities, towns, census-designated places and unincorporated communities, Hamilton County forms a region that combines both urban and rural areas. Its racial makeup is about 74.75% White and 25.25% other races. With age-adjusted death rates in the region 13.7% higher than national averages, and with the prevalence of chronic heart disease, chronic obstructive pulmonary disease, diabetes, and obesity exceeding national rates, a significant portion of the population in Hamilton County is considered highly vulnerable to COVID-19 [19]. Through a collaboration with the Chattanooga COVID-19 Data and Analytics Working Group [20], the authors of this work have been provided and continuously updated with detailed epidemic, demographic and health data for Hamilton County.

We implement our mode for Hamilton County in the time period from December 1st, 2020 to February 28th, 2021. After almost a year since COVID-19 was first reported, the general public already have a good understanding of the disease risk and get used to the social distancing normal. We thus assume that there was no significant change of human behavior during this three-month period, which allows the parameters, particularly the transmission rates, in our model to be reasonably approximated as constants [21]. On the other hand, December 2020 marked a time when the second wave of COVID-19 was spreading throughout the US. Our modeling study allows us to investigate the development and progression of this epidemic wave, taking into account the underlying health conditions of the host population, and make near-term predictions of the future evolution of COVID-19.

The remainder of this paper is organized as follows. Section 2 presents the mathematical formulation of our two-group model. Section 3 discusses parameter values, with a focus on the estimation of the transmission rates through data fitting. Section 4 conducts a sensitivity analysis to the model parameters in terms of the state variables and the basic reproduction number. Section 5 presents simulation results and near-term forecasts for the epidemic progression of COVID-19. Section 6 concludes the paper with some discussion.

## 2. Model formulation

We propose a mathematical model based on differential equations to investigate the transmission dynamics of COVID-19, with an emphasis on the relationship between the disease transmission and the chronic health conditions of the hosts. We divide the total human population into two groups: Group I consists of individuals without underlying health conditions, and Group II consists of individuals with at least one underlying health conditions. We partition each group *i* (*i* = 1, 2) into five compartments, including the susceptible individuals (denoted by *S*_*i*_), the exposed individuals (denoted by *E*_*i*_), the infected but non-hospitalized individuals (denoted by *I*_*i*_), the hospitalized individuals (denoted by *H*_*i*_), and the recovered individuals (denoted by *R*_*i*_).

Both the exposed and infected individuals are assumed to be infectious and are capable of transmitting the disease to susceptible individuals [22–24]. The exposed compartment in our model is treated the same as a pre-symptomatic or asymptomatic compartment in other studies [11, 25]. Individuals in the exposed compartment typically do not show symptoms and have not been tested/confirmed; they may either recover directly from the exposed state, or transfer into the infected and hospitalized states after an incubation period. Individuals in the infected compartment have tested positive but only show minor or moderate symptoms. They are typically advised, though not in a mandatory manner, to self-quarantine at home until full recovery. Individuals in the hospitalized compartment have tested positive and are at high risk that necessitates hospital admission. We assume that disease-induced deaths only occur in hospitalized individuals. We also assume that hospitalized individuals do not have contact with the public due to their strict isolation, and they will not transmit the disease to others.

**Figure 1.**
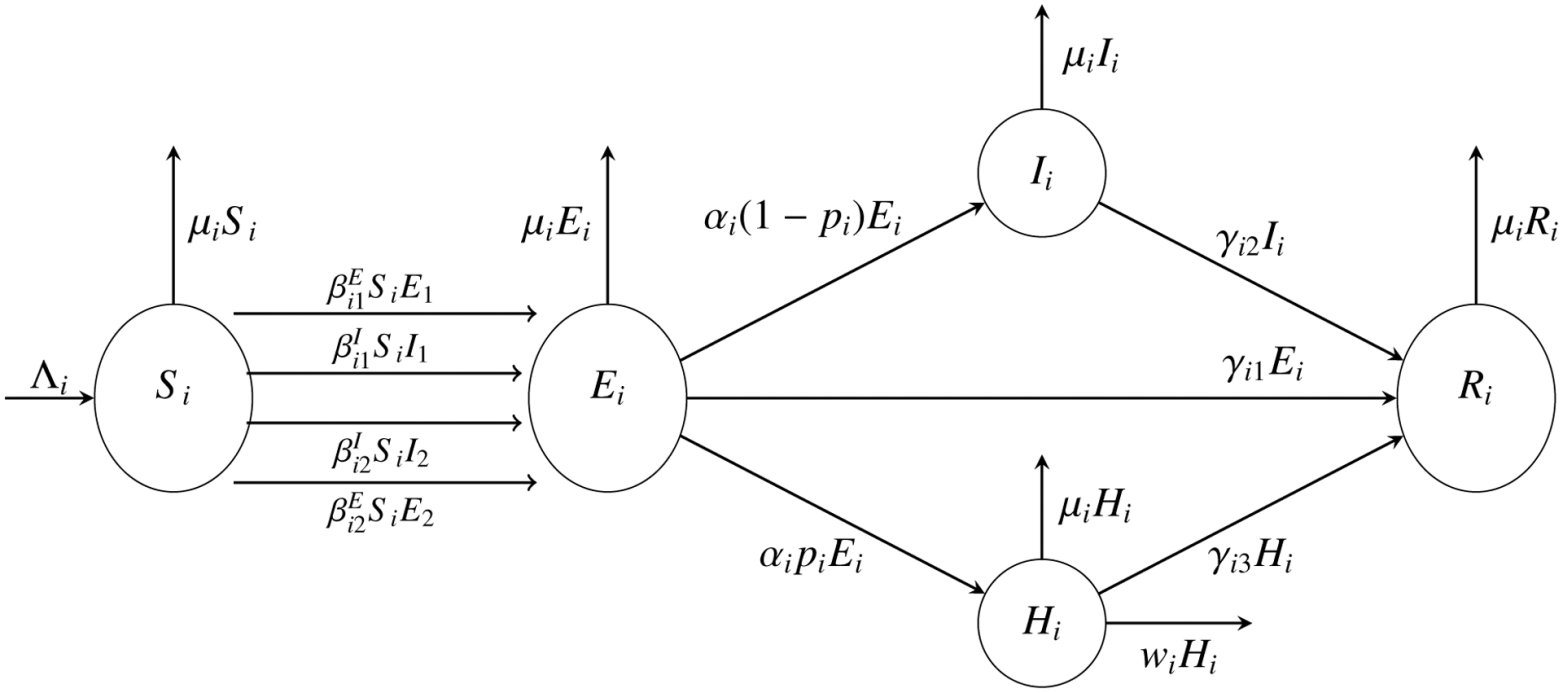
Flow diagram for Group *i* (*i* = 1, 2).

Our two-group COVID-19 model is described by the following system. A flow diagram for this model is given in Figure 1.

Group I: Individuals without underlying health conditions.

(2.1) $$\begin{array}{ll} \frac{dS_{1}}{dt} & =\Lambda_{1}-\beta_{11}^{E}S_{1}E_{1}-\beta_{11}^{I}S_{1}I_{1}-\beta_{12}^{E}S_{1}E_{2}-\beta_{12}^{I}S_{1}I_{2}-\mu_{1}S_{1},\\ \frac{dE_{1}}{dt} & =\beta_{11}^{E}S_{1}E_{1}+\beta_{11}^{I}S_{1}I_{1}+\beta_{12}^{E}S_{1}E_{2}+\beta_{12}^{I}S_{1}I_{2}-\left(\gamma_{11}+\alpha_{1}+\mu_{1}\right)E_{1},\\ \frac{dI_{1}}{dt} & =\alpha_{1}\left(1-p_{1}\right)E_{1}-\left(\gamma_{12}+\mu_{1}\right)I_{1},\\ \frac{dH_{1}}{dt} & =\alpha_{1}p_{1}E_{1}-\left(w_{1}+\gamma_{13}+\mu_{1}\right)H_{1},\\ \frac{dR_{1}}{dt} & =\gamma_{11}E_{1}+\gamma_{12}I_{1}+\gamma_{13}H_{1}-\mu_{1}R_{1}. \end{array}$$

Group II: Individuals with underlying health conditions.

(2.2) $$\begin{array}{ll} \frac{dS_{2}}{dt} & =\Lambda_{2}-\beta_{21}^{E}S_{2}E_{1}-\beta_{21}^{I}S_{2}I_{1}-\beta_{22}^{E}S_{2}E_{2}-\beta_{22}^{I}S_{2}I_{2}-\mu_{2}S_{2},\\ \frac{dE_{2}}{dt} & =\beta_{21}^{E}S_{2}E_{1}+\beta_{21}^{I}S_{2}I_{1}+\beta_{22}^{E}S_{2}E_{2}+\beta_{22}^{I}S_{2}I_{2}-\left(\gamma_{21}+\alpha_{2}+\mu_{2}\right)E_{2},\\ \frac{dI_{2}}{dt} & =\alpha_{2}\left(1-p_{2}\right)E_{2}-\left(\gamma_{22}+\mu_{2}\right)I_{2},\\ \frac{dH_{2}}{dt} & =\alpha_{2}p_{2}E_{2}-\left(w_{2}+\gamma_{23}+\mu_{2}\right)H_{2},\\ \frac{dR_{2}}{dt} & =\gamma_{21}E_{2}+\gamma_{22}I_{2}+\gamma_{23}H_{2}-\mu_{2}R_{2}. \end{array}$$

For each group *i* (*i* = 1, 2), Λ_*i*_ is the population influx rate, *μ*_*i*_ is the natural death rate, *α*_*i*_ is the incubation rate, *p*_*i*_ is the portion of exposed individuals who become severely ill and hospitalized after the incubation period, *γ*_*i*1_, *γ*_*i*2_ and *γ*_*i*3_ are the rates of recovery from the exposed, infected (non-hospitalized), and hospitalized individuals, respectively, and *w*_*i*_ is the disease-induced death rate. The parameters $\beta_{ij}^{E}$ and $\beta_{ij}^{I}$ (*i*, *j* = 1, 2) are the transmission rates between compartments *S*_*i*_ and *E*_*j*_, and between compartments *S*_*i*_ and *I*_*j*_, respectively. We assume that all these parameters are constants, and their values are discussed in the next section.

## 3 Data fitting

We list the model parameters and their base values in Table 1. For those parameters whose base values are not available, we will use data fitting to estimate their values. The total population of the region in this study (Hamilton County) is *N* = 367, 804 [18]. According to an estimate from CDC [5], about 40% of the population have at least one underlying health conditions. We thus set the population sizes of the two groups as *N*_1_ = 0.6*N* and *N*_2_ = 0.4*N*. We calculate the influx rate of susceptible individuals in each group (*i* = 1, 2) by Λ_*i*_ = *μ*_*i*_*N*_*i*_, where we take *μ*_1_ = *μ*_2_ = *μ* as the natural birth and death rate in the region. The incubation period of the infection ranges between 2 and 14 days, with a mean of 5–7 days [26]. We choose the base value of $\alpha_{1}^{-1}=\alpha_{2}^{-1}=7$ days in our model. Among individuals who have tested positive, the portion of severe infections, which would lead to hospitalization, ranges from 5% to 20% [27]. A recent study conducted by CDC found that hospitalizations were 6 times higher and deaths were 12 times higher among those with reported underlying conditions, compared to those with none reported [4]. We thus take the values *p*_1_ = 0.03, *p*_2_ = 0.18, and *w*_1_ = 1.2 × 10^−3^, *w*_2_ = 1.44 × 10^−2^. The recovery period from COVID-19 has a wide variation (1.5–30 days) among different patients [27], depending on their severities, ages, and overall health conditions. In our model, disease recovery occurs in the exposed, infected, and hospitalized compartments. Those who recover directly from the exposed state typically exhibit no symptoms and have a fast recovery; we set their average recovery period as 5 days in the model, which gives *γ*_11_ = *γ*_21_ = 0.2 per day. Most of the infected individuals, with minor or moderate symptoms, may recover without going to a hospital; we set their average recovery rates as *γ*_12_ = 0.12 per day and *γ*_22_ = 0.08 per day. The hospitalized individuals, typically with more severe symptoms, may need a longer recovery period; on the other hand, they receive intensive medical treatment which may accelerate their recovery process. Moreover, it is observed that the length of the average hospital stay for COVID-19 patients with chronic health conditions is 1.5 times that for those without underlying conditions [28]. Hence, we take their average recovery rates *γ*_13_ = 0.12 per day and *γ*_23_ = 0.08 per day as well.

Other parameter include the 8 transmission rates $\beta_{ij}^{E}$ and $\beta_{ij}^{I}$ (*i*, *j* = 1, 2), which typically vary from place to place and from time to time. Prior studies [11, 15, 16] have shown that the transmission rates are especially sensitive for COVID-19 modeling and have significant impact on the model output. In this study, we estimate all these transmission rates through data fitting, based on the regional COVID-19 data for Hamilton County [20].

**Figure 2.**
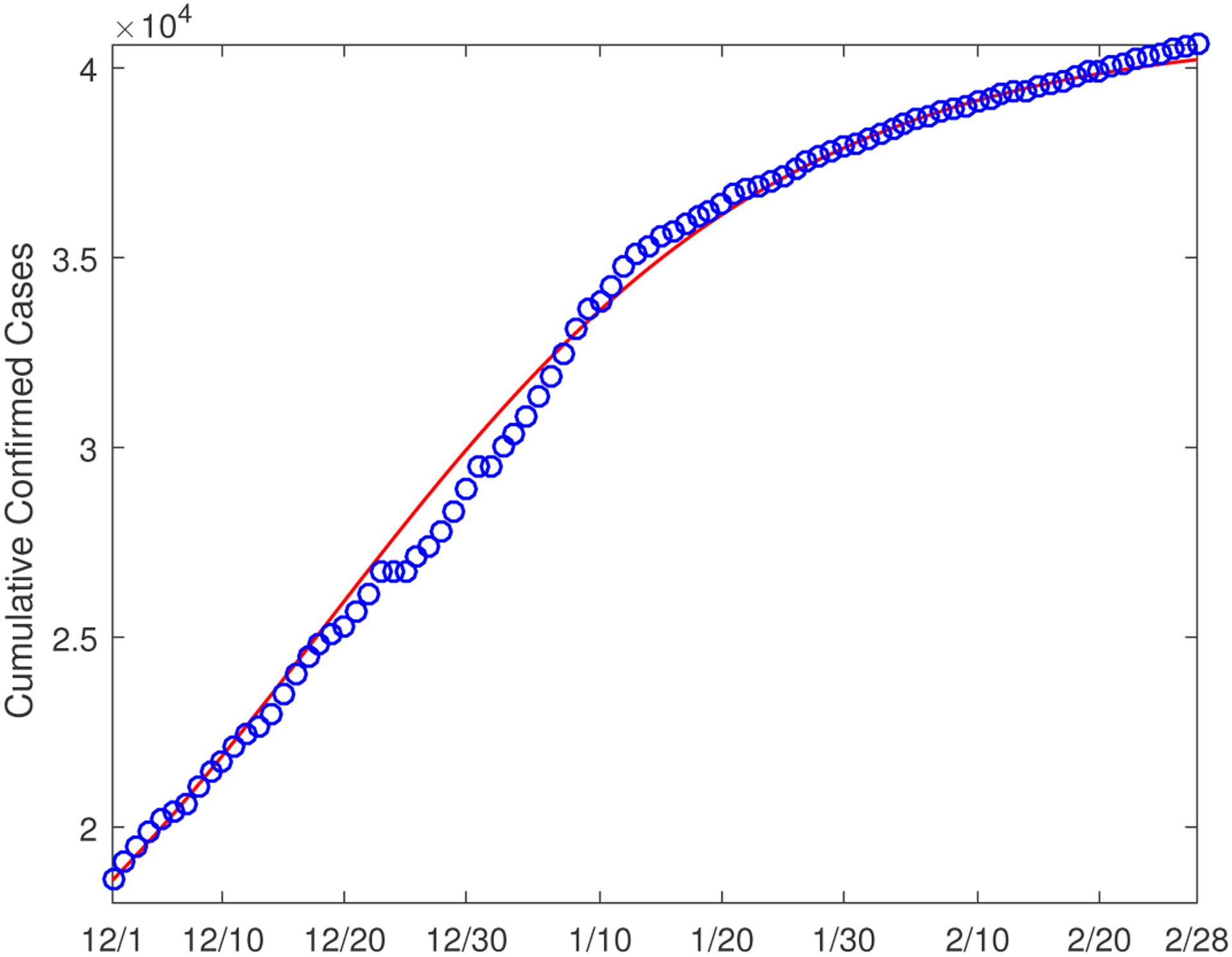
Data fitting result for the cumulative confirmed cases in Hamilton County from 12/1/2020 to 2/28/2021. The circles (in blue) denote the reported cases and the solid line (in red) denotes the fitting result.

We start our numerical study on December 1, 2020, when the second wave of COVID-19 was spreading throughout the US. We run the simulation and data fitting for a three-month period (until February 28, 2021). Using the demographic and epidemic data reported in Hamilton County [19,20], we set the initial conditions as: *S*_1_(0) = 203164, *E*_1_(0) = 2000, *I*_1_(0) = 3000, *H*_1_(0) = 18, *R*_1_(0) = 13000; *S*_2_(0) = 144315, *E*_2_(0) = 300, *I*_2_(0) = 500, *H*_2_(0) = 107, *R*_2_(0) = 2000. Figure 2 shows the reported number of cumulative confirmed cases in Hamilton County versus our fitting curve in this three-month period. We observe a high degree of match between our simulation result and the reported data. The parameter values found through the data fitting and their 95% confidence intervals (CI) are presented in Table 2.

In order to quantify the goodness-of-fit, we calculate the normalized mean square error (NMSE), which is defined by

$$\text{NMSE}=\frac{n\sum_{i=1}^{n}{\left(y_{i}-{\hat{y}}_{i}\right)}^{2}}{\left(\sum_{i=1}^{n}y_{i}\right)\left(\sum_{i=1}^{n}{\hat{y}}_{i}\right)},$$

where *y*_*i*_ (1 ≤ *i* ≤ *n*) are the reported data, ${\hat{y}}_{i}(1\leq i\leq n)$ are the simulated data, and *n* is the number of data points used. In general, a lower value of NMSE indicates a better quality of fitting. We find that the NMSE for our data fitting is 0.00023.

In Eq (A3) of the Appendix, we have derived the basic reproduction number $R_{0}$ for our model. In Theorem A1.1, we have shown that when $R_{0}<1$, the disease would be eradicated. Based on the parameter values in Tables 1 and 2, we are able to evaluate the basic reproduction number in this region, and we find that $R_{0}\approx 1.16$, which is consistent with the persistence of the disease during these three months.

In addition, we observe in Table 2 that $\beta_{11}^{E}$, $\beta_{12}^{E}$, $\beta_{21}^{E}$ and $\beta_{22}^{E}$ are much higher in values than $\beta_{11}^{I}$, $\beta_{12}^{I}$, $\beta_{21}^{I}$ and $\beta_{22}^{I}$, indicating that the exposed individuals play a significantly larger role than that of the infected individuals in the disease transmission and spread. This can be clearly expected since infected individuals who have tested positive are generally recommended or required to quarantine at home, while those severely infected ones are treated and isolated in hospitals, and so they possess a lower risk in transmitting the disease compared to the exposed individuals who are asymptomatic but infectious. Meanwhile, among the four parameters associated with the exposed-to-susceptible transmission route, we see that $\beta_{11}^{E}$ is the largest and $\beta_{22}^{E}$ is the second largest, and even the second largest one is about four times of the values of $\beta_{12}^{E}$ and $\beta_{22}^{E}$, showing that the contact and transmission within each group may play a more important role than the cross-transmission between the two groups (I and II).

## 4. Sensitivity analysis

Our model involves a relatively large number of parameters. To investigate changes of which parameters have higher impact on model output, we conduct a sensitivity analysis of the parameters with respect to the state variables and the basic reproduction number. We consider the following 20 parameters, $\beta_{11}^{E}$, $\beta_{12}^{E}$, $\beta_{21}^{E}$, $\beta_{22}^{E}$, $\beta_{11}^{I}$, $\beta_{12}^{I}$, $\beta_{21}^{I}$, $\beta_{22}^{I}$, *γ*_11_, *γ*_12_, *γ*_13_, *γ*_21_, *γ*_22_, *γ*_23_, *α*_1_, *α*_2_, *p*_1_, *p*_2_, *w*_1_, and *w*_2_, in our model. The sensitivity of the state variables measures the influence of parameters on model prediction, whereas the sensitivity of the basic reproduction number quantifies the influence of parameters in shaping the disease risk.

We first employ the basic differential equation analysis approach [29] to derive the sensitivity equations for our system (2.1) and (2.2). Denote the sets of state variables by

$$X_{1}=\left\{S_{1},E_{1},I_{1},H_{1},R_{1}\right\},\;X_{2}=\left\{S_{2},E_{2},I_{2},H_{2},R_{2}\right\},$$

and the set of parameters by

$$P=\left\{\beta_{11}^{E},\beta_{12}^{E},\beta_{21}^{E},\beta_{22}^{E},\beta_{11}^{I},\beta_{12}^{I},\beta_{21}^{I},\beta_{22}^{I},\gamma_{11},\gamma_{12},\gamma_{13},\gamma_{21},\gamma_{22},\gamma_{23},\alpha_{1},\alpha_{2},p_{1},p_{2},w_{1},w_{2}\right\}.$$

For $X_{i}\in X_{i}$ and *y* ∈ *P*, we define the relative sensitivity *s*(*X*_*i*_, *y*) of the state *X*_*i*_ to the parameter *y*, non-dimensionalized by the state *X*_*i*_ and the parameter value *y*, as

(4.1) $$s\left(X_{i},y\right)=\frac{\partial X_{i}}{\partial y}\cdot \frac{y}{X_{i}},\;X_{i}\neq 0,\;i=1,2.$$

To compute the partial derivative $\frac{\partial X_{i}}{\partial y}$, which is also referred to as a quasi-state variable, we differentiate it with respect to *t* to obtain

(4.2) $$\frac{\partial }{\partial t}\left(\frac{\partial X_{i}}{\partial y}\right)=\frac{\partial }{\partial y}\left(\frac{\partial X_{i}}{\partial t}\right),\;X_{i}\in X_{i},\;y\in P,\;i=1,2.$$

We then numerically solve for the quasi-state solutions $\left\{\frac{\partial X_{i}}{\partial y}:X_{i}\in X_{i},y\in P,\;i=1,2\right\}$ by associating systems (2.1) and (2.2) with system (4.2).

**Figure 3.**
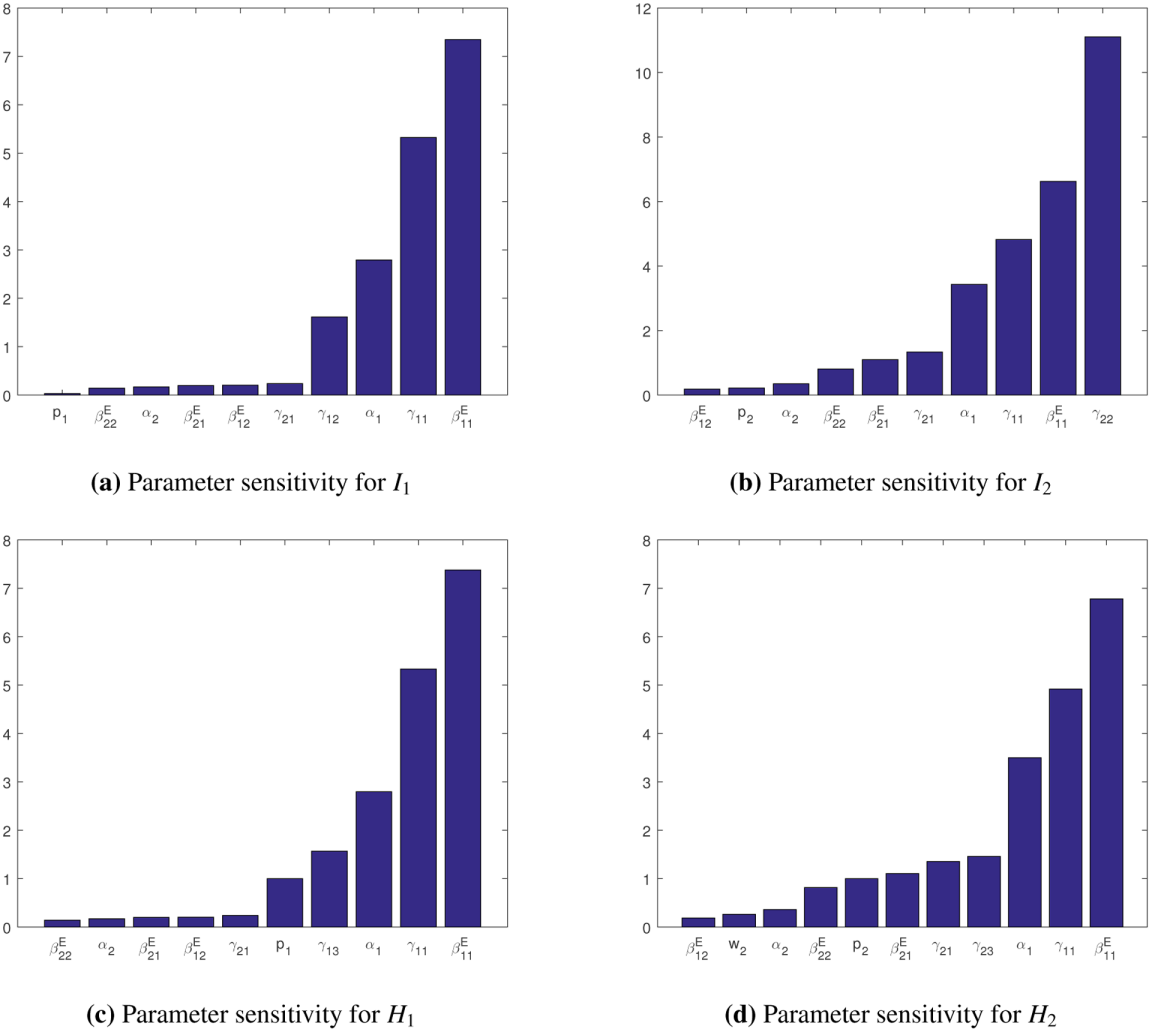
Relative sensitivities of the most sensitive parameters for the numbers of infected and hospitalized individuals.

A typical set of results are presented in Figure 3, where we list the relative sensitivities of the state variables *H*_1_, *H*_2_, *I*_1_ and *I*_2_ with respect to the most sensitive parameters in the set *P*. Unlisted parameters have low sensitivities that are very close to 0. We clearly observe that $\beta_{11}^{E}$ has the highest sensitivity for *H*_1_, *H*_2_ and *I*_1_, and the second highest sensitivity for *I*_2_, which implies that the exposed-to-susceptible transmission route within Group I has a major impact on the epidemic progression. Meanwhile, we see the other three parameters associated with the exposed-to-susceptible transmission route; i.e., $\beta_{12}^{E}$, $\beta_{21}^{E}$ and $\beta_{22}^{E}$, are also sensitive for all the four state variables, consistent with our observation from the data fitting result. Additionally, we find that the incubation rates (represented by *α*_*i*_), recovery rates (represented by *γ*_*ij*_), and hospitalization ratios (represented by *p*_*i*_), are also among the sensitive parameters for the four state variables. We will further explore the impact of these sensitive parameters on the simulation results in section 5.

Next, we use the expression in equation (A3) to compute the relative sensitivity of the basic reproduction number $R_{0}$ with respect to each parameter *y* ∈ *P*; i.e., $\frac{\partial R_{0}}{\partial y}\cdot \frac{y}{R_{0}}$. The results are listed in Table 3, where the parameters are ranked in terms of their sensitivities. We observe a general pattern consistent with that in Figure 3. In particular, we observe that the four transmission rates $\beta_{11}^{E}$, $\beta_{22}^{E}$, $\beta_{12}^{E}$ and $\beta_{21}^{E}$ have the highest sensitivity for $R_{0}$, indicating that the changes of their values would have most significant impact on the reduction of the basic reproduction number. According to Theorem A1.1, reducing $R_{0}$ below unity would eradicate the infection. Consequently, disease control measures reducing the contact rate (such as social distancing) or the transmission probability (such as vaccination) between the exposed and susceptible individuals, may be most efficient in containing the COVID-19 epidemic.

**Figure 4.**
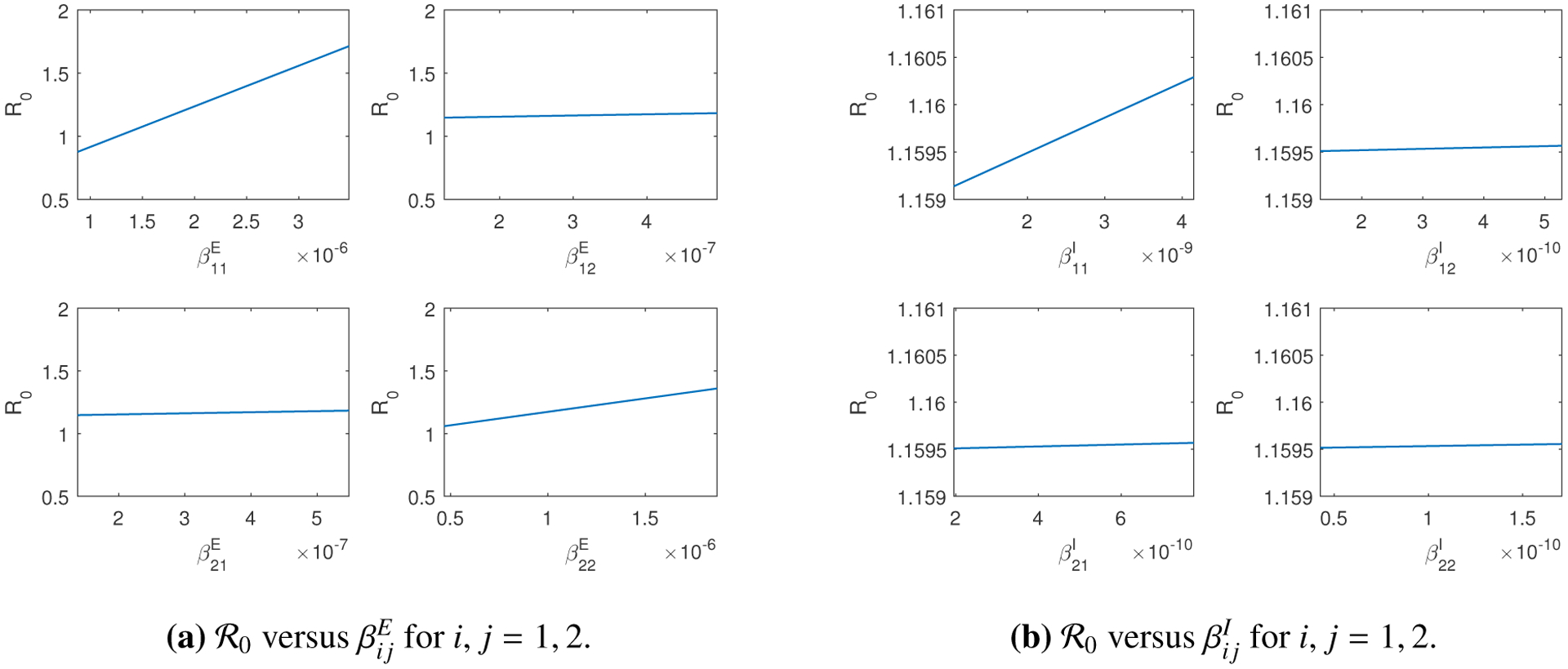
Variations of $R_{0}$ in terms of the transmission rates.

Moreover, we visualize the variations of $R_{0}$ with respect to each transmission rate in Figure 4. Specifically, we change the value of each transmission rate from 50% to 200% of its base value in Table 2, and use Eq (A3) to calculate $R_{0}$ correspondingly. Figures 4a and 4b again show that $R_{0}$ is typically more sensitive to $\beta_{ij}^{E}$ than to $\beta_{ij}^{I}$, *i*, *j* = 1,2, which is another piece of evidence that exposed individuals play a greater role than that of infected individuals in the disease transmission and spread.

## 5. Simulation results

**Figure 5.**
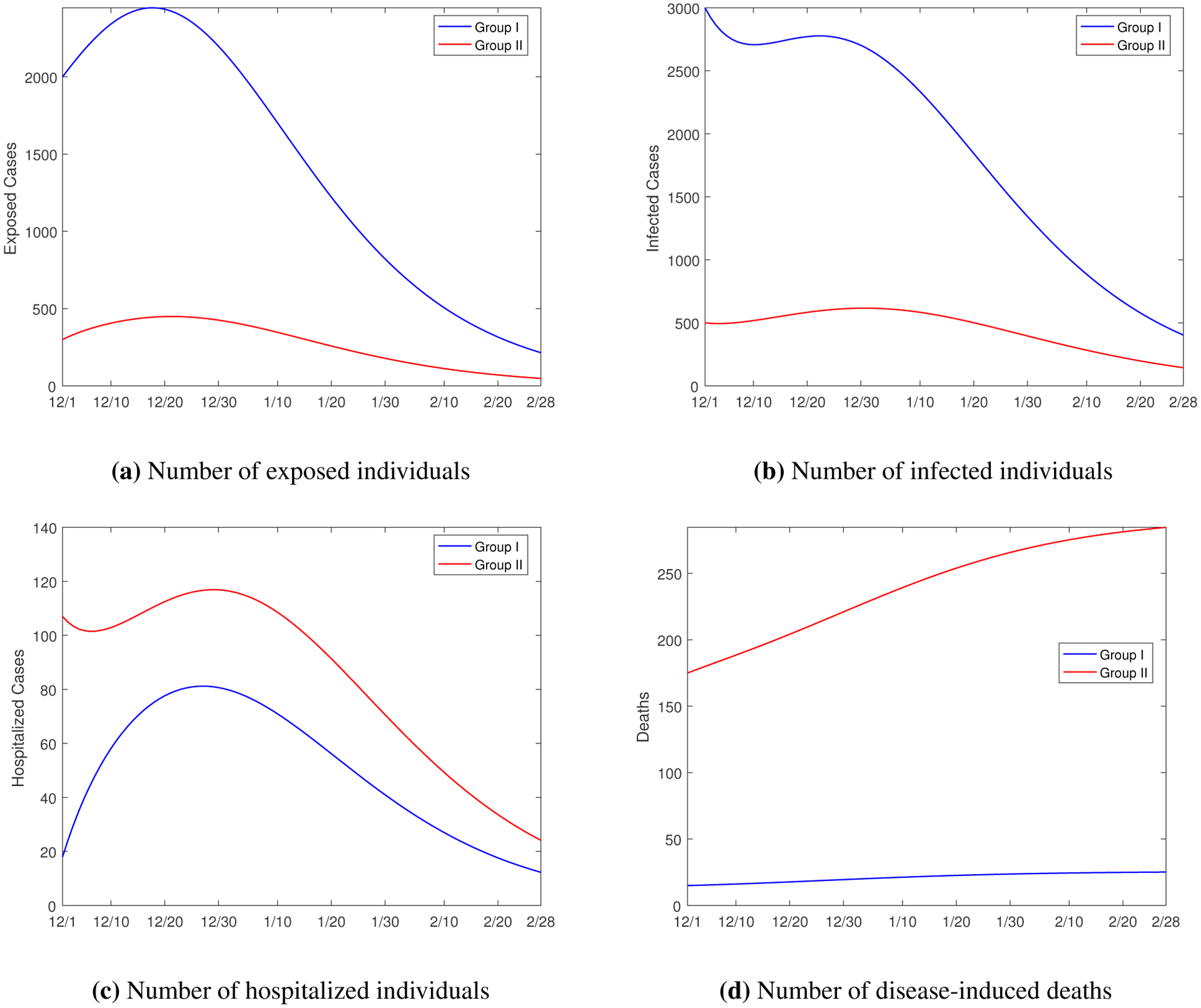
Simulation results for the numbers of exposed, infected and hospitalized individuals in Group I (without underlying health conditions) and Group II (with underlying health conditions).

Our data fitting and numerical simulation are conducted on the three-month period from December 1, 2020 to February 28, 2021. Figure 5 displays the exposed cases, infected cases, and hospitalized cases in Group I (without underlying health conditions) and Group II (with underlying health conditions). We observe that the numbers of exposed and infected individuals in Group I are much higher than those in Group II (see Figure 5a,b). These differences can be possibly explained by: (1) the size of Group I is larger than that of Group II; (2) individuals in Group I, considered as more healthy, generally have a higher level of physical activity, including mobility and personal contact, than that for individuals in Group II; and (3) individuals in Group II, aware of their underlying health conditions, are generally more cautious about the infection risk of COVID-19. On the other hand, Figure 5c,d shows that the numbers of hospitalizations and deaths in Group I are significantly lower than those in Group II, since individuals with underlying health conditions have a much higher chance to develop severe illness due to COVID-19. In particular, the number of disease-induced deaths in Group I only increased slightly and remained at a very low level during the three-month period , whereas the number of deaths in Group II increased by more than 100 in the same period. For the hospitalized cases, the curves for both groups reach a peak around December 30, several days behind the peaks of the exposed and infected cases, and then decline afterwards. The time interval for the occurrence of the peak values of the exposed, infected and hospitalized cases in both groups coincide with the Christmas–New Year holiday period, a reflection of the impact of the increased mobility and personal contact due to holiday travels.

**Figure 6.**
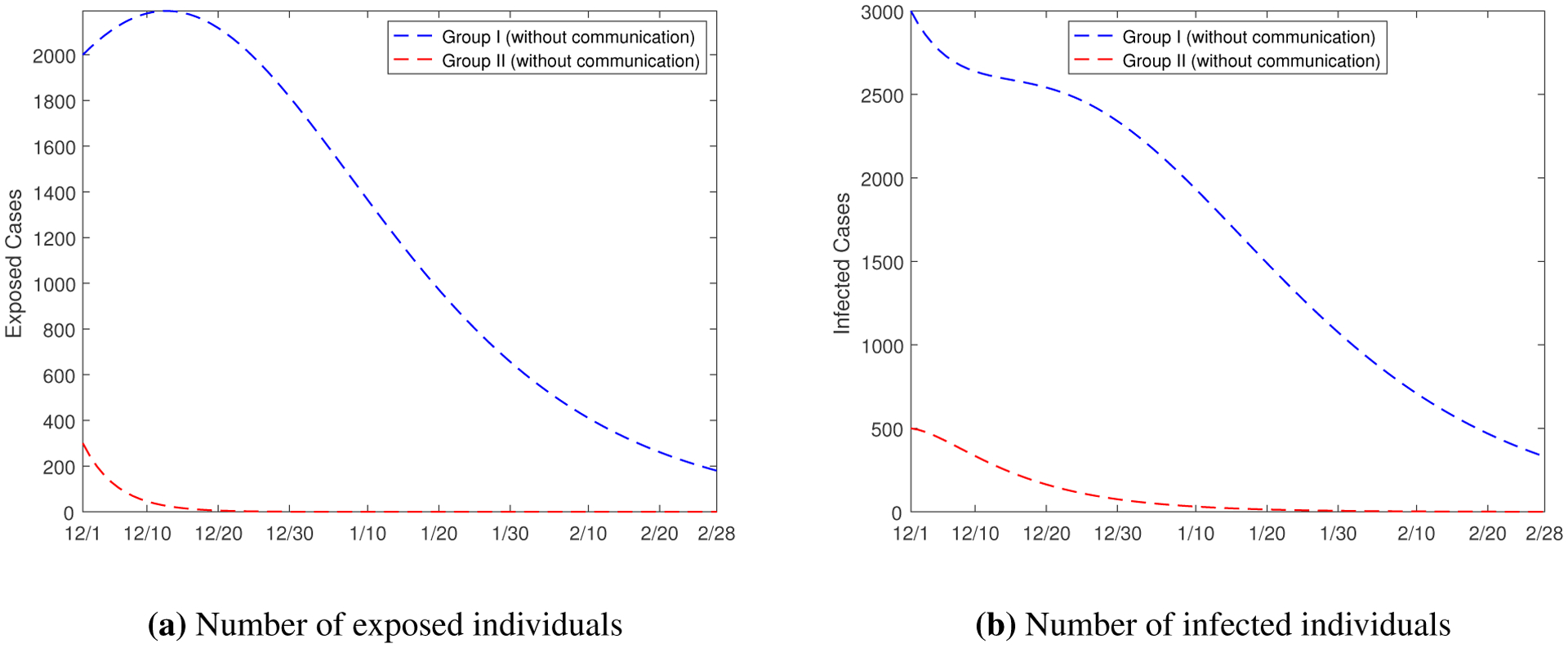
Simulation results for the numbers of exposed and infected individuals in a hypothetical scenario where there is no communication between Group I and Group II.

Figure 5 provides base scenarios of our model simulation from December 1, 2020 to February 28, 2021, using transmission rates estimated from data fitting that are presented in Table 2. Also, from the same table, we have observed that the values of the within-group transmission rates $\beta_{11}^{E}$ and $\beta_{22}^{E}$ are much higher than those of the inter-group transmission rates $\beta_{12}^{E}$ and $\beta_{21}^{E}$. In order to quantify the role played by the cross-transmission between the two groups, we simulate a hypothetical scenario where there is no communication between the two groups; i.e., the two groups are decoupled from each other. Effectively, we set the four between-group transmission rates $\beta_{12}^{E}$, $\beta_{21}^{E}$, $\beta_{12}^{I}$ and $\beta_{21}^{I}$ to zero, and run the model simulation. Results for the numbers of exposed and infected individuals are presented in Figure 6. Compared to Figures 5a and 5b, we see that the curves for Group I only have slight changes, whereas the curves for Group II are dramatically different. Without the between-group transmission, the numbers of exposed and infected individuals in Group II would both quickly approach zero. This pattern implies that the cross-transmission has a minor effect on the Group I disease dynamics, but it is critical for the disease progression and persistence in Group II. Neglecting such cross-transmission would severely underestimate the disease risk for Group II.

**Figure 7.**
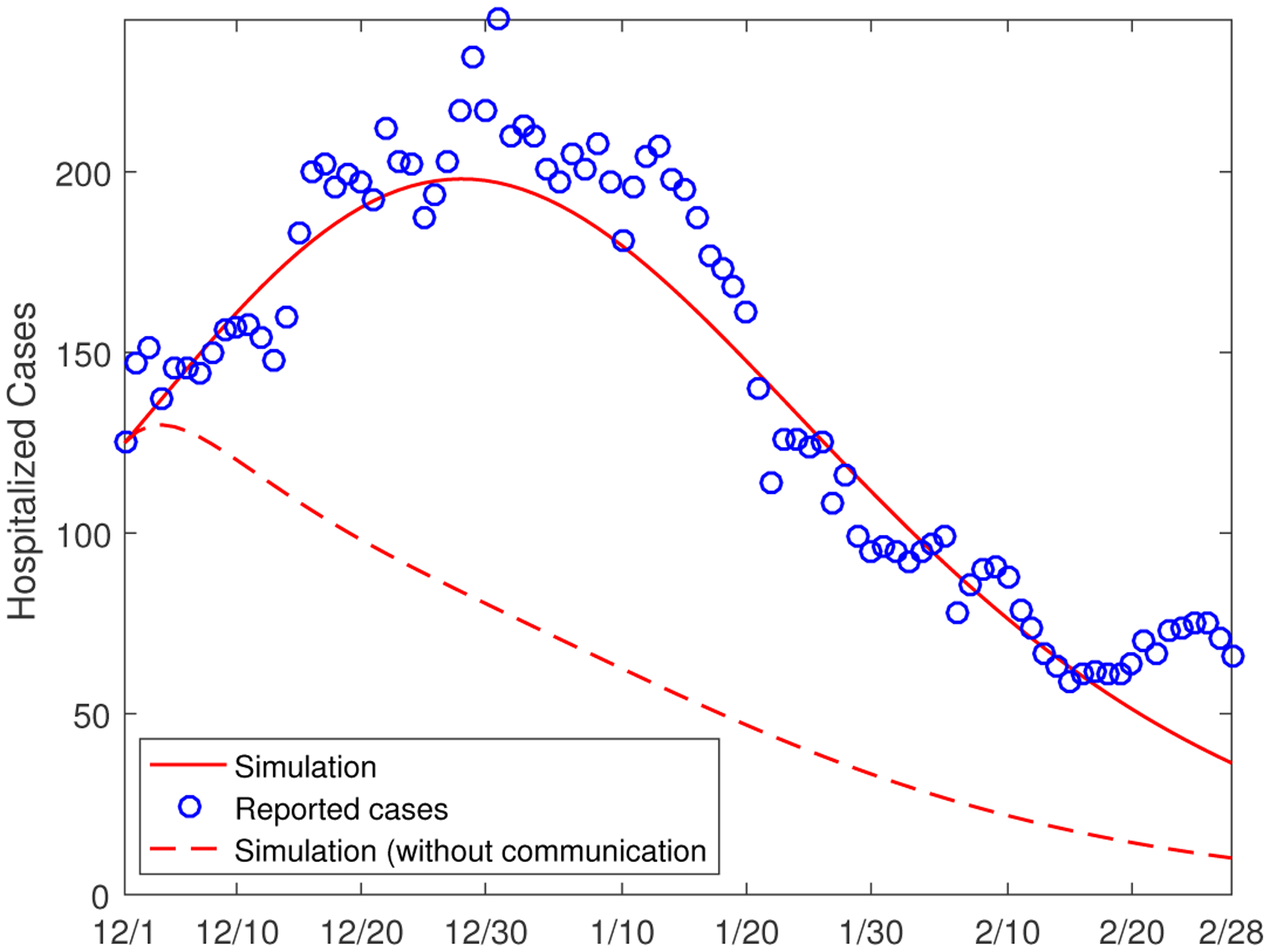
Comparison between the reported number of hospitalizations and the numerical simulation results. The circles (in blue) represent the reported cases, the solid line (in red) represents the simulation result from the original model, and the dashed line (in red) represents the simulation result for the scenario where the between-group communication is not present.

A major concern of the health administrations is whether the hospital capacity can meet the demands of COVID-19 patients with severe illness. This underscores the importance of accurate simulation and prediction of hospitalizations that result from the COVID-19 infection. Our model is capable of computing the number of hospitalized cases from each of the two population groups. As a means to validate our model simulation, we calculate the total number of hospitalizations; i.e., *H*_1_ + *H*_2_ in our model, and compare with the reported hospitalized cases from December 1, 2020 to February 28, 2021. Figure 7 depicts this comparison, and we observe a similar trend and reasonably good agreement between these two sets of (reported and simulated) data. In particular, we notice that the peak values of the hospitalized cases, for both the reported and simulated data, occur around December 30, similar to what we observed in Figure 5c. Additionally, we have also plotted in Figure 7 the simulation result for the hypothetical scenario where the inter-group transmission is removed, and we again observe a significant underestimate for the number of hospitalizations.

The results in Figures 6 and 7 imply that, from the disease control point of view, reducing the between-group contact could be a strategic approach to bring down the exposed, infected and hospitalized cases in Group II, and to effectively protect the individuals with underlying health conditions.

**Figure 8.**
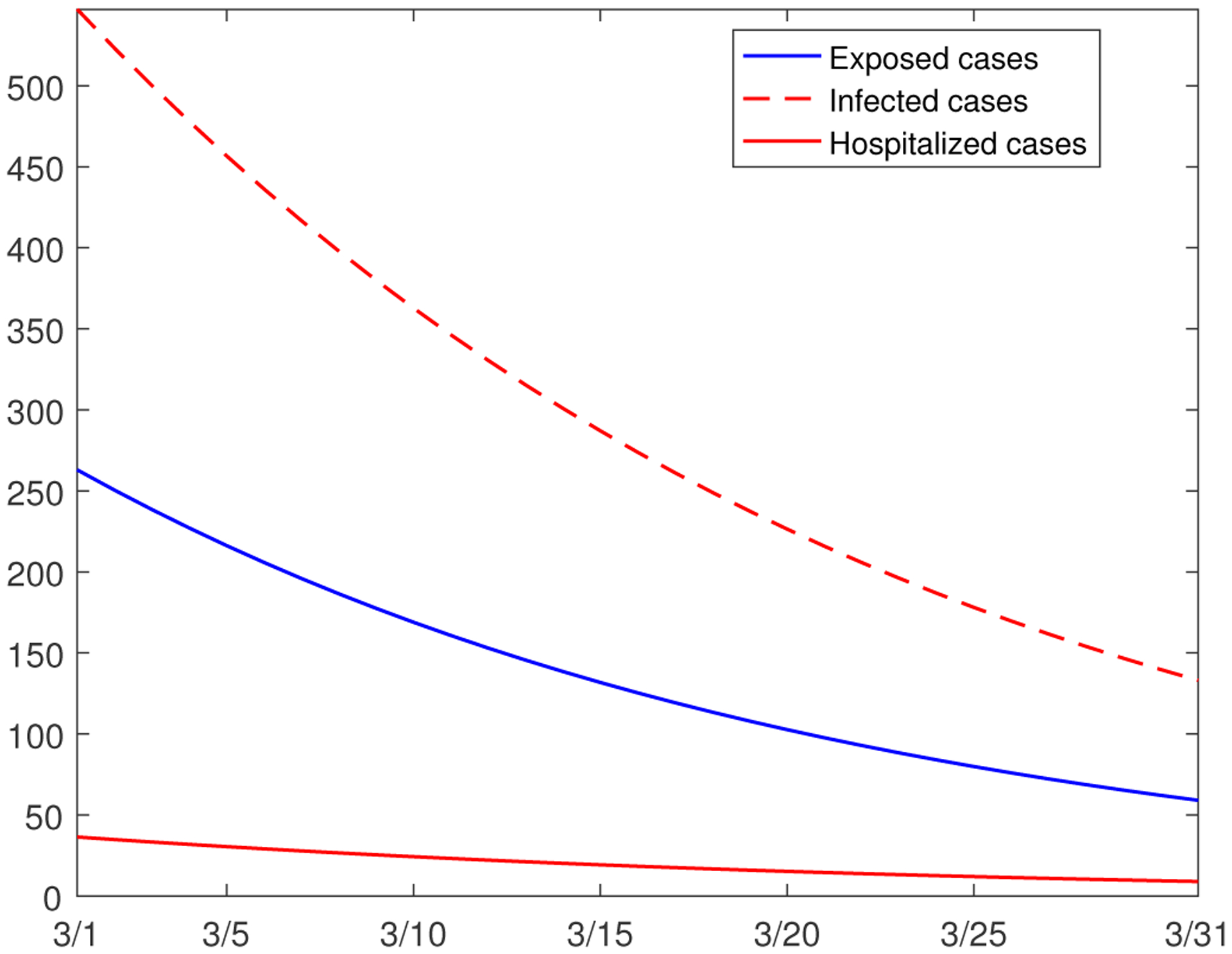
Simulation results for the numbers of exposed, infected, and hospitalized individuals from March 1 to March 31, 2021.

In what follows, we use our model to make predictions for the near future with regard to COVID-19 epidemic development in Hamilton County. Figure 8 shows the simulation results for the numbers of exposed, infected, and hospitalized cases (two groups combined) for the one-month period from March 1, 2021 to March 31, 2021, based on the parameter values in Tables 1 and 2. We clearly see that all the three curve move downward, a continuation of the decline of the epidemic from the previous two months (see Figure 5). In particular, the decrease of the infected cases is substantial.

Figure 8 is regarded as a base scenario for our model prediction in the near term (March 1 to March 31, 2021). Since our model involves many parameters, and since some of these parameters are considerably sensitive (see section 4 for our sensitivity analysis results), we perform a detailed simulation study to quantify the changes of the model predictions when the values of these most sensible parameters vary.

**Figure 9.**
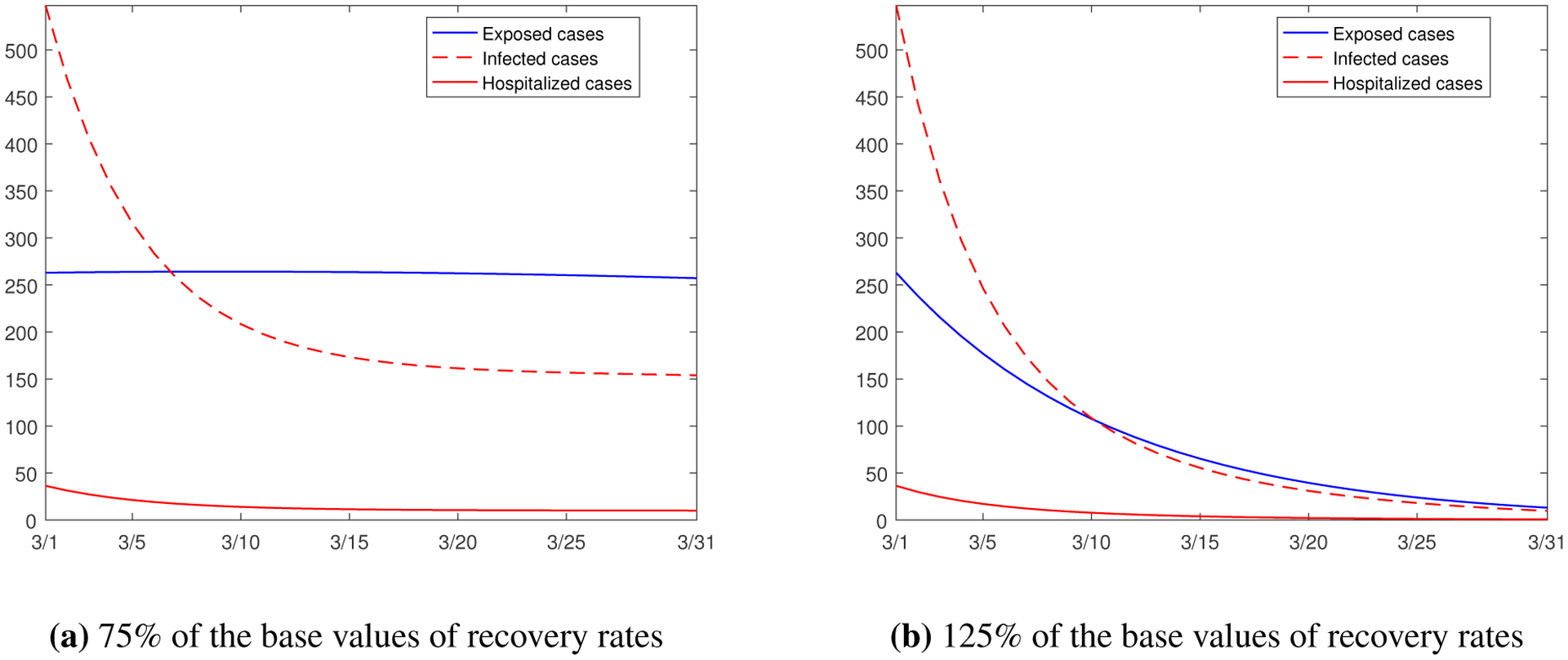
Simulation results for the numbers of the exposed, infected, and hospitalized individuals from March 1 to March 31, 2021, with different recovery rates *γ*_*ij*_ (*i* = 1, 2; *j* = 1, 2, 3).

We first study the variation of the recovery rates. The parameters *γ*_*i*1_, *γ*_*i*2_ and *γ*_*i*3_ in our model represent the recovery rates of the exposed, infected and hospitalized individuals, respectively, in group *i* (*i* = 1, 2). We consider a scenario where each recovery rate is reduced to 75% of its base value, and present the simulation result for the same period in Figure 9a. In comparison with Figure 8, we see that the decline of the exposed and infected cases slows down in Figure 9a, while there is little change to the number of hospitalizations. Meanwhile, we consider another scenario where each recovery rate is increased to 125% of its base value, and present the simulation result in Figure 9b. We see that the numbers of the exposed, infected and hospitalized individuals all decrease much faster, compared with Figure 8. The variation of recovery rates could be caused by factors such as the change of environmental conditions, the improvement of medical care standards in the region, the evolution of the immunity level in the host population, and the mutation of the viral strains. The results in Figure 9 demonstrate that higher (lower) recover rates would accelerate (slow down) the elimination of the epidemic.

**Figure 10.**
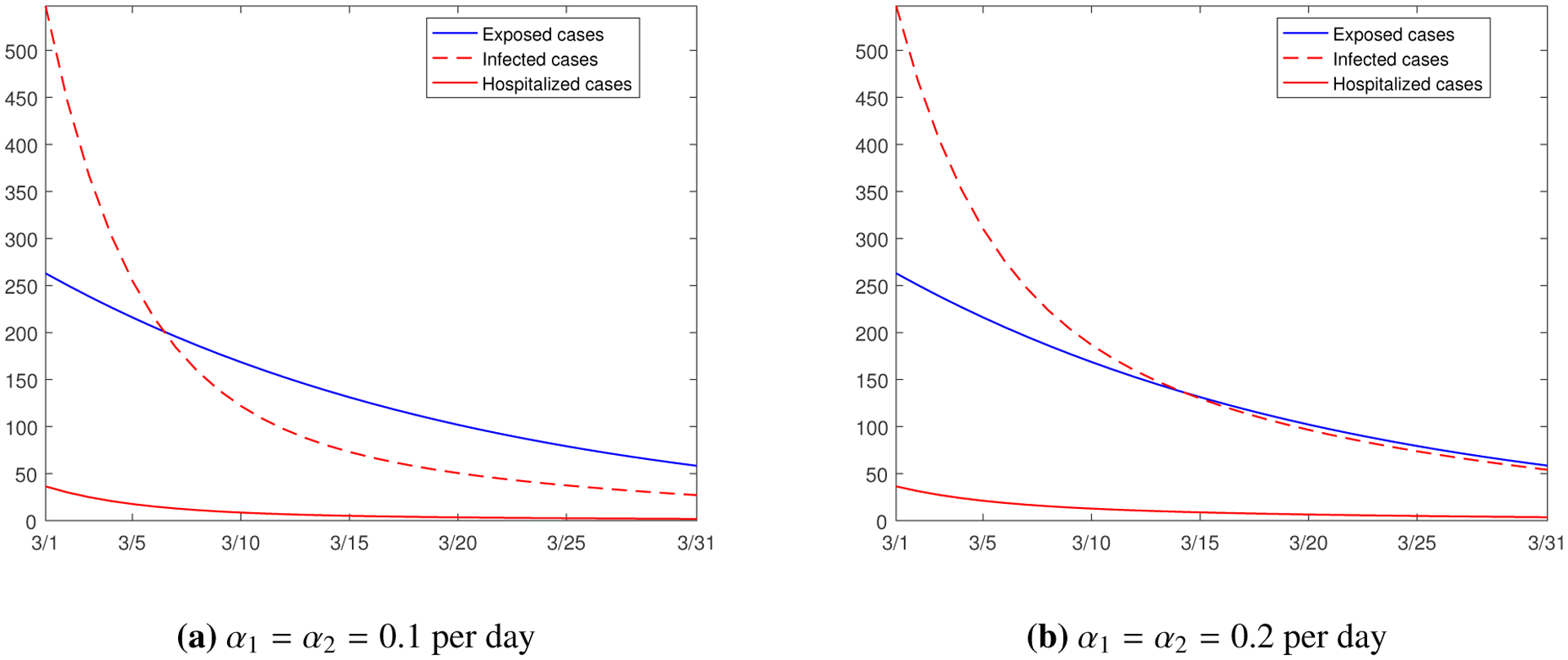
Simulation results for the numbers of the exposed, infected, and hospitalized individuals from March 1 to March 31, 2021, with different incubation rates represented by *α*_*i*_ (*i* = 1, 2).

We also consider the impact of the incubation periods on the epidemic progression. The parameters *α*_*i*_ in our model represent the incubation rate (i.e., the reciprocal of the incubation length) in group *i* (*i* = 1, 2), and their base values are *α*_1_ = *α*_2_ = 1/7 per day. Similar to recovery rates, the incubation rates could change due to the health conditions of the hosts and the characteristics of the coronavirus. Figure 10 shows the simulation results with decreased incubation rates *α*_1_ = *α*_2_ = 0.1 per day, and increased rates *α*_1_ = *α*_2_ = 0.2 per day, while other parameters are all fixed. Comparing Figures 10a and 10b, we see that there is little difference for the numbers of exposed and hospitalized individuals, while the impact is more significant on the number of infected individuals: larger values of incubation rates correspond to shorter incubation periods, resulting in a stronger influx into the infected class which leads to a higher level of infection.

**Figure 11.**
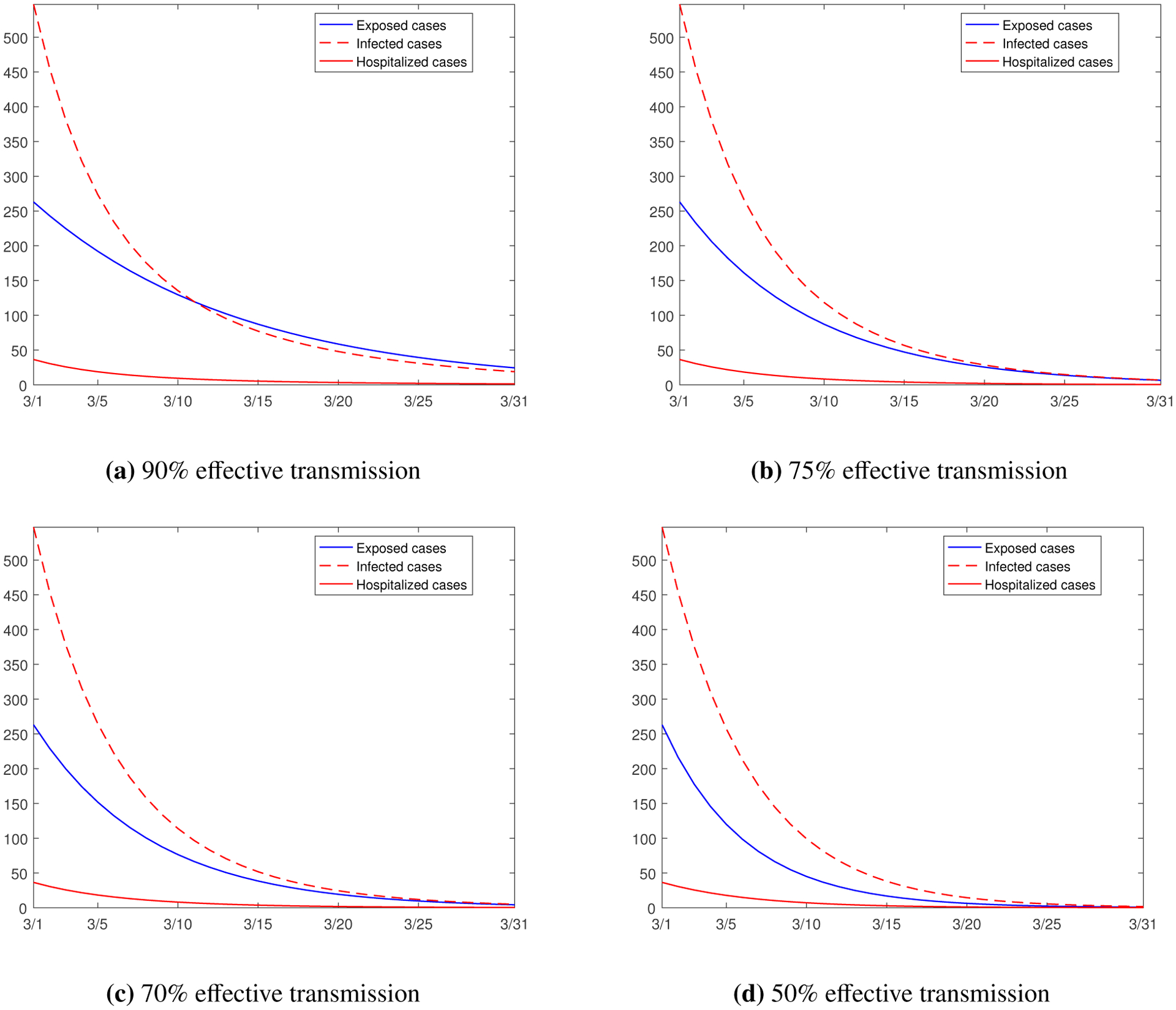
Simulation results for the numbers of the exposed, infected, and hospitalized individuals from March 1 to March 31, 2021, with reduced transmission rates represented by 90%, 75%, 70% and 50% of the base values.

Next, we consider the variation of the transmission rates. As discussed in previous sections, the transmission rates are sensitive to both the state variables and the basic reproduction number. The COVID-19 vaccination campaign is currently on-going throughout the US, with over 2 million shots administered each day. For Hamilton County, about 15.5% of the total population had been at least partially vaccinated as of March 5, 2021 [30]. As the vaccination coverage quickly increases, the probability of human hosts contracting the coronavirus will decrease, which will effectively reduce the disease transmission rates. Here we consider three possible scenarios where all the transmission rates are reduced to 90%, 75%, and 70%, respectively, and another (more hypothetical) scenario where all the transmission rates are reduced to 50%, of their respective base values given in Table 2. In other words, we assume that the disease transmission would be only 90%, 75%, 70% and 50% effective, respectively, during the month of March 2021, compared to that in previous three months. The simulation results are presented in Figure 11. As can be naturally expected, the reduction of transmission rates quickly brings down the numbers of the exposed, infected, and hospitalized individuals, and the curves all approach zero in the more hypothetical case with only 50% effective transmission.

**Figure 12.**
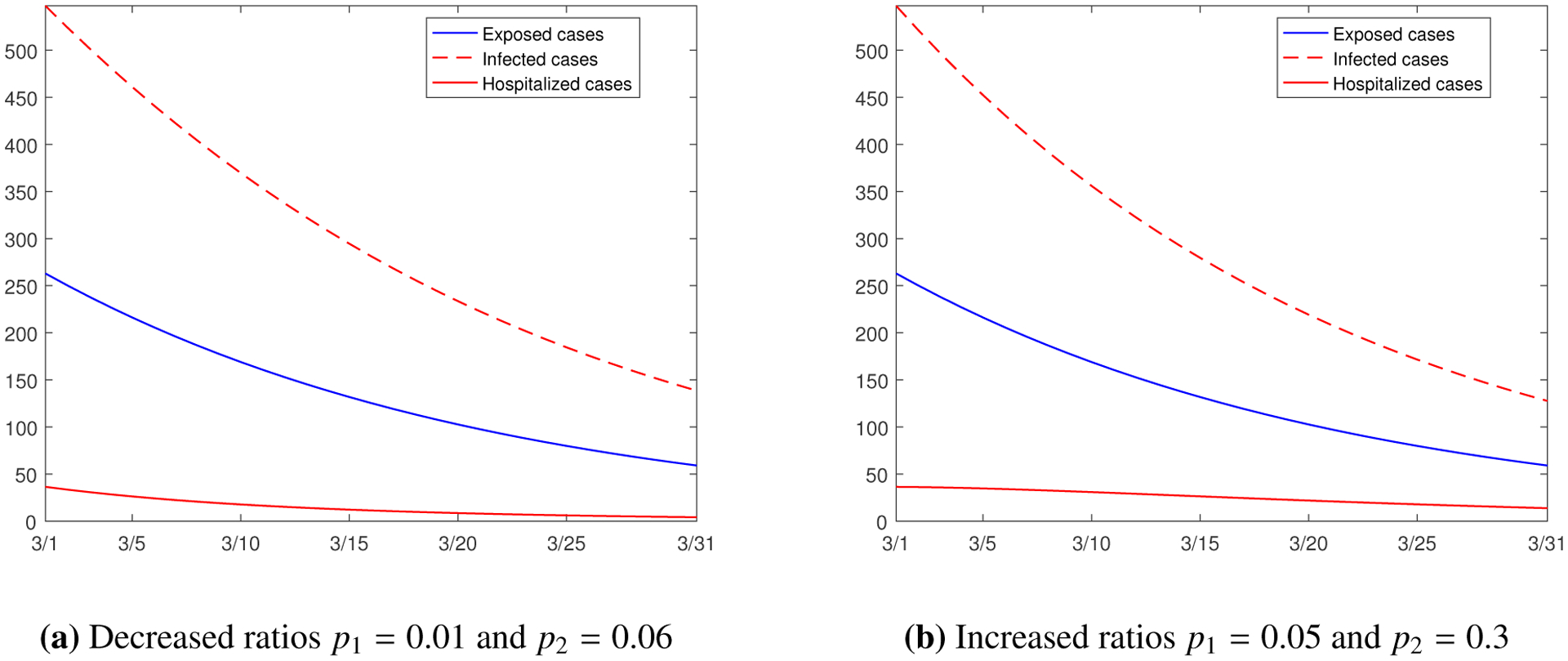
Simulation results for the numbers of the exposed, infected, and hospitalized individuals from March 1 to March 31, 2021, with different values of hospitalization ratios *p*_1_ and *p*_2_.

In addition, we examine the changes of the hospitalization ratios *p*_1_ and *p*_2_ and their impact on the model prediction. The base values are *p*_1_ = 0.03 and *p*_2_ = 0.18 in our model. Figure 12a depicts the simulation result for decreased hospitalization ratios *p*_1_ = 0.01 and *p*_2_ = 0.06, and Figure 12b depicts the simulation result for increased hospitalization ratios *p*_1_ = 0.05 and *p*_2_ = 0.3. The two sets of results shows very little difference for the exposed and infected cases, while it is noticeable that the hospitalized cases decline faster with the reduced hospitalization ratios, which could be possibly achieved through the on-going vaccination campaign that places individuals with underlying health conditions into a priority group.

**Figure 13.**
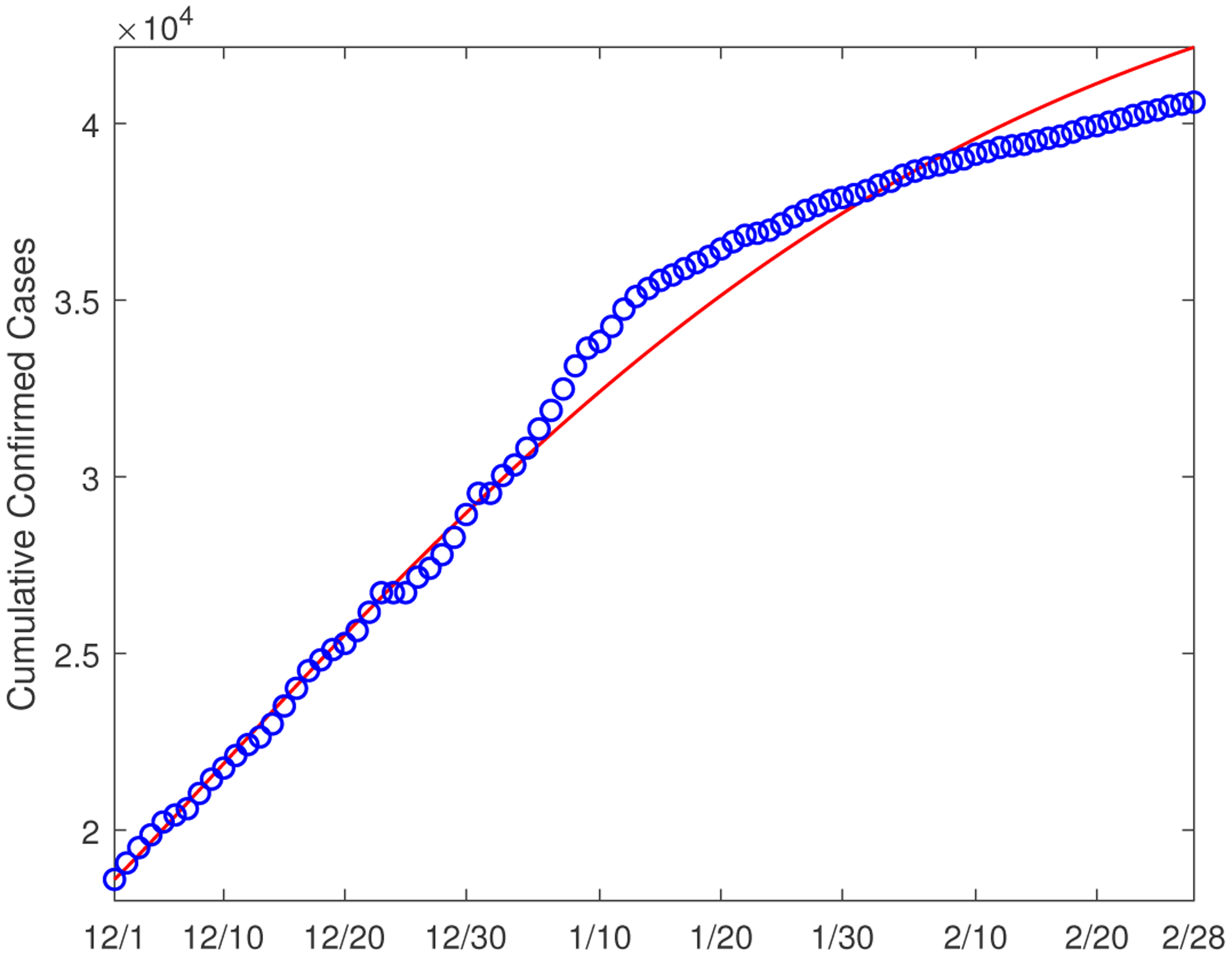
Data fitting result for the cumulative confirmed cases in Hamilton County from 12/1/2020 to 2/28/2021 based on the infected-to-susceptible transmission only. The circles (in blue) denote the reported cases and the solid line (in red) denotes the fitting result.

Finally, we discuss another modeling scenario concerned with the disease transmission by exposed individuals. In our model system (2.1) and (2.2), a person in the exposed compartment *E* is essentially regarded as a pre-symptomatic or asymptomatic infectious individual who can directly transmit COVID-19 to susceptible people [22–25]. For comparison, we now assume that exposed individuals in compartment *E* are latent and not capable of transmitting the disease [31]. To that end, we remove the exposed-to-susceptible transmission route by setting the transmission rates $\beta_{ij}^{E}=0$ (*i*, *j* = 1, 2) in system (2.1) and (2.2). In this way we obtain a two-group system where each group allows only the infected-to-susceptible transmission route and is more like a traditional SEIR model. We then conduct data fitting to estimate the four transmission rates $\beta_{ij}^{I}$ (*i*, *j* = 1, 2) using the same reported data from December 1, 2020 to February 28, 2021. The fitting curve for the cumulative cases is shown in Figure 13. The normalized mean square error (NMSE) for this data fitting is 0.00071, in comparison to 0.00023 for our original model fitting (see Figure 2). The parameter values found through the fitting and their 95% confidence intervals are presented in Table 4. Based on these values, we find that the basic reproduction number in this case is given by $R_{0}\approx 1.12$, which is comparable to our estimate of 1.16 for the original model (see section 3).

**Figure 14.**
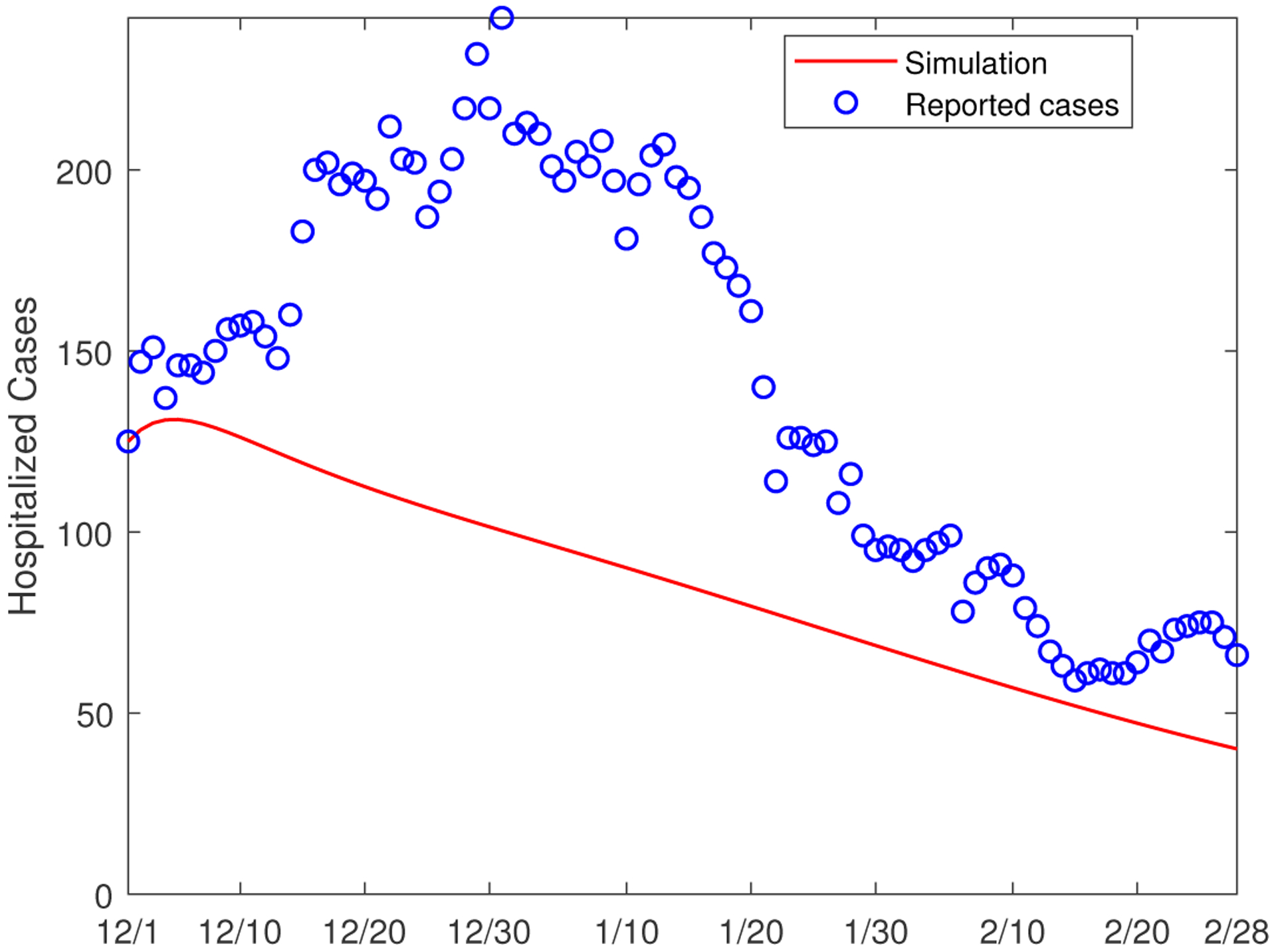
Comparison between the reported number of hospitalizations in Hamilton County and the numerical simulation result based on the infected-to-susceptible transmission only. The circles (in blue) represent the reported cases, and the solid line (in red) represents the simulation result.

Using the parameter values from this data fitting, we numerically calculate the total number of hospitalizations, and the simulation result versus the reported hospitalized cases in the three-month period (from December 1, 2020 to February 28, 2021) are presented in Figure 14. We observe that the modified model significantly underestimates the hospitalized cases, in comparison to Figure 7 where the simulation result for the original model is represented in the solid line. Since the hospitalized individuals are mainly those with underlying health conditions, this result indicates that neglecting the exposed-to-susceptible transmission route would underrate the infection risk for the more vulnerable population group (i.e., Group II). The finding appears to be consistent with our previous observations, including the sensitivity analysis in section 4, that the transmission rates $\beta_{ij}^{E}$ (*i*, *j* = 1, 2) play an important role in shaping the overall transmission pattern and infection risk. Hence, our original model (2.1)(2.2) seems to be a better choice in addressing the correlation between COVID-19 transmission and underlying medical conditions, the main goal of this study.

## 6. Conclusions

We have presented a new mathematical model to investigate the relationship between the transmission and spread of COVID-19 and the underlying health conditions of the host population. The model divides the population into two groups based on the presence/absence of chronic conditions, and incorporates the transmission of the disease both within and between groups. As a demonstration of our model application, we have performed a case study for Hamilton County in the US state of Tennessee, a typical place with high prevalence of chronic health conditions.

With all the transmission rates estimated by parameter fitting based on the regional data, we have conducted a detailed numerical investigation on the numbers of exposed, infected and hospitalized cases that come from individuals with and without chronic conditions. Our simulation results agree well with the reported data. We have also conducted extensive simulations when a number of sensitive parameters change in values, the results of which help us to better understand the progression and evolution of COVID-19 in the near future.

Our simulation results quantify and confirm the high risk of individuals with chronic conditions. Specifically, the population group with underlying health conditions constantly produces much higher numbers of hospitalizations and deaths, compared to the group without underlying conditions. Our findings highlight the importance of weakening the disease transmission route between the exposed and susceptible individuals, for both the population groups, in fighting COVID-19. Social distancing, which reduces the personal contact, and vaccination deployment, which reduces the transmission probability, would both be critical approaches to achieve this goal. In particular, we find that reducing the between-group contact is effective in protecting the vulnerable group against the COVID-19 infection, and this control strategy seems to be productive in bringing down the numbers of infections and hospitalizations for the group with chronic conditions.

The model output predicts a general decline of the COVID-19 epidemic in the near future for the region in this study, even with the variation of several sensitive and important parameters. In particular, the on-going vaccination campaign is expected to continue improving the immunity level in the host population, particularly for those with chronic conditions, and speed up the process of containing the epidemic.

## Figures and Tables

**Table 1.** Model parameters and their base values (p=person, d=day).

Parameter	Description	Value	Source
*N*	Population size in Hamilton County	367804 p	[18]
*μ*	Natural birth and death rate	2.74 × 10^−5^/d	[19]
*α*_1_	Incubation rate in group I	1/7/d	[26]
*α*_2_	Incubation rate in group II	1/7/d	[26]
*p*_1_	Ratio of hospitalization in group I	0.03	[4, 27]
*p*_2_	Ratio of hospitalization in group II	0.18	[4, 27]
*w*_1_	Disease-induced death rate in group I	0.0012/d	[2, 4]
*w*_2_	Disease-induced death rate in group II	0.0144/d	[2, 4]
*γ*_11_	Recovery rate of exposed individuals in group I	0.2/d	[27]
*γ*_21_	Recovery rate of exposed individuals in group II	0.2/d	[27]
*γ*_12_	Recovery rate of infected individuals in group I	0.12/d	[27, 28]
*γ*_22_	Recovery rate of infected individuals in group II	0.08/d	[27, 28]
*γ*_13_	Recovery rate of hospitalized individuals in group I	0.12/d	[27, 28]
*γ*_23_	Recovery rate of hospitalized individuals in group II	0.08/d	[27, 28]
$\beta_{11}^{E}$	Transmission rate between *S*_1_ and *E*_1_	fitting by data	–
$\beta_{12}^{E}$	Transmission rate between *S*_1_ and *E*_2_	fitting by data	–
$\beta_{21}^{E}$	Transmission rate between *S*_2_ and *E*_1_	fitting by data	–
$\beta_{22}^{E}$	Transmission rate between *S*_2_ and *E*_2_	fitting by data	–
$\beta_{11}^{I}$	Transmission rate between *S*_1_ and *I*_1_	fitting by data	–
$\beta_{12}^{I}$	Transmission rate between *S*_1_ and *I*_2_	fitting by data	–
$\beta_{21}^{I}$	Transmission rate between *S*_2_ and *I*_1_	fitting by data	–
$\beta_{22}^{I}$	Transmission rate between *S*_2_ and *I*_2_	fitting by data	–

**Table 2.** Parameter values estimated by data fitting.

Parameter	Fitted value	95% CI	Parameter	Fitted value	95% CI
$\beta_{11}^{E}$	1.77 × 10^−6^	(0, 3.25 × 10^−5^)	$\beta_{11}^{I}$	2.10 × 10^−9^	(0, 5.04 × 10^−5^)
$\beta_{12}^{E}$	2.50 × 10^−7^	(0, 3.13 × 10^−4^)	$\beta_{12}^{I}$	2.63 × 10^−10^	(0, 1.71 × 10^−5^)
$\beta_{21}^{E}$	2.76 × 10^−7^	(0, 5.67 × 10^−4^)	$\beta_{21}^{I}$	3.91 × 10^−10^	(0, 3.44 × 10^−5^)
$\beta_{22}^{E}$	9.36 × 10^−7^	(0, 1.35 × 10^−4^)	$\beta_{22}^{I}$	8.55 × 10^−11^	(0, 1.04 × 10^−6^)

**Table 3.** Ranked parameter sensitivity (from the highest to the lowest) for $R_{0}$.

Rank	Parameter	Sensitivity	Rank	Parameter	Sensitivity
1	$\beta_{11}^{E}$	0.828	11	*γ*_22_	3.12 × 10^−5^
2	$\beta_{22}^{E}$	5.26 × 10^−2^	12	$\beta_{21}^{I}$	2.56 × 10^−5^
3	$\beta_{12}^{E}$	1.57 × 10^−2^	13	$\beta_{12}^{I}$	2.42 × 10^−5^
4	$\beta_{21}^{E}$	1.56 × 10^−2^	14	*α*_2_	1.82 × 10^−5^
5	*γ*_12_	1.17 × 10^−3^	15	$\beta_{22}^{I}$	7.04 × 10^−6^
6	$\beta_{11}^{I}$	1.14 × 10^−3^	16	*p*_2_	6.85 × 10^−6^
7	*α*_1_	6.81 × 10^−4^	17	*γ*_13_	0
8	*γ*_11_	6.80 × 10^−4^	18	*γ*_23_	0
9	*γ*_21_	1.82 × 10^−4^	19	*w*_1_	0
10	*p*_1_	3.61 × 10^−5^	20	*w*_2_	0

**Table 4.** Infected-to-susceptible transmission rates estimated by data fitting.

Parameter	Fitted value	95% Confidence Interval
$\beta_{11}^{I}$	1.53 × 10^−6^	(1.38 × 10^−8^, 3.05 × 10^−6^)
$\beta_{12}^{I}$	1.36 × 10^−7^	(0, 3.67 × 10^−6^)
$\beta_{21}^{I}$	3.30 × 10^−8^	(0, 5.40 × 10^−7^)
$\beta_{22}^{I}$	3.23 × 10^−10^	(0, 2.57 × 10^−8^)

## Appendix

### A1. Basic reproduction number

We derive the basic reproduction number for the proposed two-group COVID-19 model. Note that in the original system (2.1) and (2.2), the compartments *S*_*i*_, *E*_*i*_, and *I*_*i*_ do not depend on the compartments *H*_*i*_ and *R*_*i*_ (*i* = 1, 2). We can thus combine the two subsystems (2.1) and (2.2) and study the following reduced system instead.

(A1) $$\begin{array}{ll} \frac{dS_{1}}{dt} & =\Lambda_{1}-\beta_{11}^{E}S_{1}E_{1}-\beta_{11}^{I}S_{1}I_{1}-\beta_{12}^{E}S_{1}E_{2}-\beta_{12}^{I}S_{1}I_{2}-\mu_{1}S_{1},\\ \frac{dE_{1}}{dt} & =\beta_{11}^{E}S_{1}E_{1}+\beta_{11}^{I}S_{1}I_{1}+\beta_{12}^{E}S_{1}E_{2}+\beta_{12}^{I}S_{1}I_{2}-\left(\gamma_{11}+\alpha_{1}+\mu_{1}\right)E_{1},\\ \frac{dI_{1}}{dt} & =\alpha_{1}\left(1-p_{1}\right)E_{1}-\left(\gamma_{12}+\mu_{1}\right)I_{1},\\ \frac{dS_{2}}{dt} & =\Lambda_{2}-\beta_{21}^{E}S_{2}E_{1}-\beta_{21}^{I}S_{2}I_{1}-\beta_{22}^{E}S_{2}E_{2}-\beta_{22}^{I}S_{2}I_{2}-\mu_{2}S_{2},\\ \frac{dE_{2}}{dt} & =\beta_{21}^{E}S_{2}E_{1}+\beta_{21}^{I}S_{2}I_{1}+\beta_{22}^{E}S_{2}E_{2}+\beta_{22}^{I}S_{2}I_{2}-\left(\gamma_{21}+\alpha_{2}+\mu_{2}\right)E_{2},\\ \frac{dI_{2}}{dt} & =\alpha_{2}\left(1-p_{2}\right)E_{2}-\left(\gamma_{22}+\mu_{2}\right)I_{2}. \end{array}$$

It is easy to verify that the system (A1) has a unique disease-free equilibrium (DFE) at

(A2) $$x_{0}=\left(S_{1}^{0},E_{1}^{0},I_{1}^{0},S_{2}^{0},E_{2}^{0},I_{2}^{0}\right)=\left(\frac{\Lambda_{1}}{\mu_{1}},0,0,\frac{\Lambda_{2}}{\mu_{2}},0,0\right)\text{. }$$

Based on the next-generation matrix technique [32], the new infection matrix *F* and the transition matrix *V* are given by

$$F=\left[\begin{array}{cccc} \beta_{11}^{E}S_{1}^{0} & \beta_{11}^{I}S_{1}^{0} & \beta_{12}^{E}S_{1}^{0} & \beta_{12}^{I}S_{1}^{0}\\ 0 & 0 & 0 & 0\\ \beta_{21}^{E}S_{2}^{0} & \beta_{21}^{I}S_{2}^{0} & \beta_{22}^{E}S_{2}^{0} & \beta_{22}^{I}S_{2}^{0}\\ 0 & 0 & 0 & 0 \end{array}\right]$$

and

$$V=\left[\begin{array}{cccc} u_{11} & 0 & 0 & 0\\ -\alpha_{1}\left(1-p_{1}\right) & u_{12} & 0 & 0\\ 0 & 0 & u_{21} & 0\\ 0 & 0 & -\alpha_{2}\left(1-p_{2}\right) & u_{22} \end{array}\right],$$

where *u*_*i*1_ = *γ*_i1_ + *α*_*i*_ + *μ*_*i*_ and *u*_*i*2_ = *γ*_*i*2_ + *μ*_*i*_ for *i* = 1, 2. The basic reproduction number $R_{0}$ is defined as the spectral radius of the next-generation matrix

$$FV^{-1}=\left[\begin{array}{cccc} a_{11} & \frac{\beta_{11}^{I}S_{1}^{0}}{u_{12}} & a_{12} & \frac{\beta_{12}^{I}S_{1}^{0}}{u_{22}}\\ 0 & 0 & 0 & 0\\ a_{21} & \frac{\beta_{21}^{I}S_{1}^{0}}{u_{12}} & a_{22} & \frac{\beta_{22}^{I}S_{2}^{0}}{u_{22}}\\ 0 & 0 & 0 & 0 \end{array}\right],$$

where

$$\begin{array}{l} a_{11}=\frac{\beta_{11}^{E}S_{1}^{0}}{u_{11}}+\frac{\alpha_{1}\left(1-p_{1}\right)\beta_{11}^{I}S_{1}^{0}}{u_{11}u_{12}},\;a_{12}=\frac{\beta_{12}^{E}S_{1}^{0}}{u_{21}}+\frac{\alpha_{2}\left(1-p_{2}\right)\beta_{12}^{I}S_{1}^{0}}{u_{21}u_{22}},\\ a_{21}=\frac{\beta_{21}^{E}S_{2}^{0}}{u_{11}}+\frac{\alpha_{1}\left(1-p_{1}\right)\beta_{21}^{I}S_{2}^{0}}{u_{11}u_{12}},\;a_{22}=\frac{\beta_{22}^{E}S_{2}^{0}}{u_{21}}+\frac{\alpha_{2}\left(1-p_{2}\right)\beta_{22}^{I}S_{2}^{0}}{u_{21}u_{22}}. \end{array}$$

Hence we have

(A3) $$R_{0}=\rho \left(FV^{-1}\right)=\frac{a_{11}+a_{22}+\sqrt{{\left(a_{11}-a_{22}\right)}^{2}+4a_{12}a_{21}}}{2},$$

which provides a measurement for the disease risk.

### A2. Disease-free equilibrium

The disease-free equilibrium has a special importance in an epidemic model. Mathematically, it represents a stationary state where there is no infection; practically, it represents the eventual goal of disease control measures: to eliminate the infection. We establish the following result for our two-group COVID-19 model, which indicates that if the basic reproduction number is reduced below unity, then the DFE is globally attractive; i.e., the disease would be eradicated.

**Theorem A1.1**. *If $R_{0}<1$, the DFE of system* (A1) *is globally asymptotically stable in*

$$\Omega =\left\{\left(S_{1},E_{1},I_{1},S_{2},E_{2},I_{2}\right)\in {\mathbb{R}}_{+}^{6}:S_{1}+E_{1}+I_{1}\leq S_{1}^{0},S_{2}+E_{2}+I_{2}\leq S_{2}^{0}\right\}.$$

*Proof*. Apparently, Ω is a positively invariant set for system (A1). Let **X** = (*E*_1_, *I*_1_, *E*_2_, *I*_2_)^*T*^. It is easy to observe that

$$\frac{dX}{dt}\leq (F-V)X.$$

Since $R_{0}=\rho \left(FV^{-1}\right)=\rho \left(V^{-1}F\right)$ and *V*^−1^*F* is a positive matrix, then by Perron Theorem, *V*^−1^*F* has a positive left eigenvector **u** corresponding to the eigenvalue $R_{0}$; i.e., $uV^{-1}F=R_{0}u$. Consider the following Lyapunov function

$$L=uV^{-1}X.$$

Differentiating L along the solutions of (A1), we have

(A4) $$\frac{dL}{dt}=uV^{-1}\frac{dX}{dt}\leq uV^{-1}(F-V)X=\left(R_{0}-1\right)uX.$$

Clearly, if $R_{0}<1$, the equality $\frac{dL}{dt}=0$ implies that **uX** = 0 by Eq (A4). Hence **X** = 0 and thereby $S_{1}=S_{1}^{0}$, *E*_1_ = 0, *I*_1_ = 0, $S_{2}=S_{2}^{0}$, *E*_2_ = 0, and *I*_2_ = 0. Thus, the largest invariant set on which $\frac{dL}{dt}=0$ consists of only the singleton $x_{0}=\left(S_{1}^{0},0,0,S_{2}^{0},0,0\right)$. By LaSalle’s Invariance Principle [33], the DFE *x*_0_ is globally asymptotically stable in Ω if $R_{0}<1$. ■
